# *N*-[Bis(dimethyl­amino)­methyl­idene]-2-[(triphenyl­meth­yl)sulfan­yl]ethanaminium hexa­fluoro­phosphate

**DOI:** 10.1107/S1600536811014929

**Published:** 2011-04-29

**Authors:** Adam Neuba, Ulrich Flörke, Gerald Henkel

**Affiliations:** a Department Chemie, Fakultät für Naturwissenschaften, Universität Paderborn, Warburgerstrasse 100, D-33098 Paderborn, Germany

## Abstract

The mol­ecular structure of the title compound, C_26_H_32_N_3_S^+^·PF_6_^−^, shows a protonated guanidyl group bridged by an ethyl­ene linker with a tritylsulfanyl unit. The guanidinium (gua) unit displays charge delocalization over the three N—C_gua_ bonds. The N—C—C—S group shows a folded nonplanar conformation with a torsion angle of 158.4 (1)°. In the crystal, the cation and anion are linked by an N—H⋯F inter­action.

## Related literature

For the synthesis, see: Herres-Pawlis *et al.* (2005 ▶). For related structures, see: Flörke *et al.* (2006 ▶); Neuba *et al.* (2007*c* ▶); Pruszynski *et al.* (1992 ▶). For related chemistry literature, see: Börner *et al.* (2007 ▶, 2009 ▶); Galezowski *et al.* (1994 ▶); Harmjanz (1997 ▶); Herres *et al.* (2005 ▶); Herres-Pawlis *et al.* (2009 ▶); Neuba (2009 ▶); Neuba *et al.* (2007*a* ▶,*b* ▶, 2008*a* ▶,*b* ▶, 2010 ▶, 2011 ▶); Peters *et al.* (2008 ▶); Pohl *et al.* (2000 ▶); Raab *et al.* (2003 ▶); Schneider (2000 ▶); Waden (1999 ▶); Wittman (1999 ▶); Wittmann *et al.* (2001 ▶).
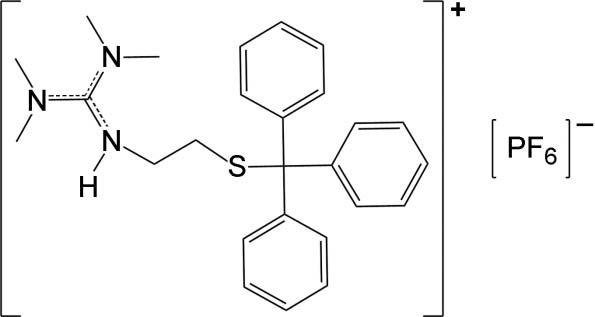


## Experimental

### 

#### Crystal data

C_26_H_32_N_3_S^+^·PF_6_^−^
*M* *_r_* = 563.58
Triclinic, 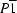

*a* = 9.0111 (14) Å
*b* = 9.1376 (15) Å
*c* = 17.564 (3) Å
α = 96.532 (3)°
β = 100.225 (4)°
γ = 108.053 (3)°
*V* = 1331.0 (4) Å^3^
*Z* = 2
Mo *K*α radiation
μ = 0.25 mm^−1^
*T* = 120 K
0.33 × 0.30 × 0.26 mm

#### Data collection

Bruker SMART APEX diffractometer
Absorption correction: multi-scan (*SADABS*; Sheldrick, 2004 ▶) *T*_min_ = 0.924, *T*_max_ = 0.939
11922 measured reflections
6281 independent reflections
4547 reflections with *I* > 2σ(*I*)
*R*_int_ = 0.060

#### Refinement

*R*[*F* ^2^ > 2σ(*F* ^2^)] = 0.047
*wR*(*F* ^2^) = 0.104
*S* = 0.96
6281 reflections
338 parameters
H-atom parameters constrained
Δρ_max_ = 0.38 e Å^−3^
Δρ_min_ = −0.42 e Å^−3^

### 

Data collection: *SMART* (Bruker, 2002 ▶); cell refinement: *SAINT* (Bruker, 2002 ▶); data reduction: *SAINT*; program(s) used to solve structure: *SHELXTL* (Sheldrick, 2008 ▶); program(s) used to refine structure: *SHELXTL*; molecular graphics: *SHELXTL*; software used to prepare material for publication: *SHELXTL* and local programs.

## Supplementary Material

Crystal structure: contains datablocks I, global. DOI: 10.1107/S1600536811014929/bt5504sup1.cif

Structure factors: contains datablocks I. DOI: 10.1107/S1600536811014929/bt5504Isup2.hkl

Supplementary material file. DOI: 10.1107/S1600536811014929/bt5504Isup3.cml

Additional supplementary materials:  crystallographic information; 3D view; checkCIF report

## Figures and Tables

**Table 1:** Hydrogen-bond geometry (Å, °)

*D*—H⋯*A*	*D*—H	H⋯*A*	*D*⋯*A*	*D*—H⋯*A*
N1—H1*A*⋯F6^i^	0.88	2.13	2.949 (2)	155

Symmetry code: (i) 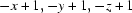.

**Fig. 1.**
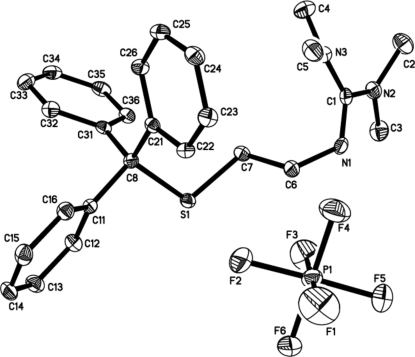
Molecular structure with displacement ellipsoids drawn at the 50% probability level. H atoms omitted for clarity.

## supplementary crystallographic information

### Comment

The synthesis and characterization of novel molecules containing nitrogen and sulfur as donor functions and their application in synthesis of sulfur copper complexes is important for biomimetic copper–sulfur chemistry. In search of multifunctional ligands we have extended our studies to guanidyl-type systems with N-donor functions. The first derivative, the ligand bis(tetramethyl-guanidino)propylene as well as amine guanidine hybrids and their complexes with Cu, Fe, Ni, Ag, Mn, Co and Zn have recently been investigated (Harmjanz, 1997; Waden, 1999; Pohl *et al.*, 2000; Schneider, 2000; Wittmann *et al.*, 2001; Herres-Pawlis *et al.*, 2005, 2009; Herres *et al.*, 2005; Neuba *et al.*, 2008*a*,*b*, 2010; Börner *et al.* 2007, 2009). We have now developed several sulfur guanidine hybrids based on aminothiophenol and cysteamine (Neuba *et al.*, 2007*a*,*b*,*c*; Neuba, 2009). The synthesized sulfur guanidine compounds possess aliphatic and aromatic thioethers or disulfide groups and were used in the synthesis of copper thiolate complexes to mimic active centres like the CuA in cytochrome-c oxidase and N_2_O-reductase (Neuba *et al.*, 2011). The title compound (I) is the protonated variant of 1,1,3,3-Tetramethyl-2-[2-(tritylsulfanyl)-ethyl]guanidine (C_26_H_31_N_3_) (Neuba *et al.*, 2007*c*). Both compounds possess a folded non-planar conformation with torsion angles of the S—C—C—N group of 66.04 (15) in C_26_H_31_N_3_ and 158.36 (13)° in I. Compared to C_26_H_32_N_3_ with localized N═C_gua_ double bond (N═C_gua_: 1.281 (2), N—C_gua_: 1.399 (2) and 1.292 (2) Å) the respective double bond in I is clearly delocalized over the guanidine unit (N═C_gua_: 1.341 (2), N—C_gua_: 1.3938 (2) and 1.333 (2) Å). Several variants of protonated bis(tetramethyl-guanidino)propylene (Flörke *et al.*, 2006) show similar N—C (1.326 (7)–1.341 (6) Å) and N—C_gua_ bond lengths (1.331 (2)–1.343 (3) Å). In bis(tetramethylguanidino)biphenyl (Pruszynski *et al.*, 1992), with a protonated imine N atom, strong delocalization is also observed among the three C—N bonds, which are in the range of 1.31 (1)-1.34 (1) Å. Further protonated guanidine units show comparable N—C_gua_– and N═C_gua_ geometries (Herres-Pawlis *et al.*, 2005; Herres *et al.*, 2005; Wittman, 1999; Peters *et al.*, 2008, Galezowski *et al.*, 1994, Raab *et al.*, 2003).

The crystal packing exhibits N1—H···F6(-*x* + 1, -*y* + 1, -*z* + 1) intermolecular interaction from cation to anion with H···F = 2.127 Å.

### Experimental

Preparation of the title compound: 1,1,3,3-Tetramethyl-2-[2-(tritylsulfanyl)-ethyl]guanidine (C_26_H_31_N_3_) (417 mg, 1 mmol) was added to a solution of [Cu(MeCN)_4_]PF_6_ (373 mg, 1 mmol) in acetonitrile (aqueous, 15 ml); the mixture was stirred for 15 min at room temperature and then refluxed for further 15 min and filtered off. Colourless crystals were obtained using the vapour pressure equalization method with this solution in the presence of diethylether.

### Refinement

H atoms were clearly identified in difference syntheses, idealized and refined riding on the C or N atoms with C—H = 0.95 (aromatic), 0.98 (methyl) and N—H 0.88 Å, and with isotropic displacement parameters *U*_iso_(H) = 1.2U(C/N_eq_) or 1.5U(–CH_3_ H atoms). All CH_3_ H atoms were allowed to rotate but not to tip.

### Crystal data

**Table:**

C_26_H_32_N_3_S^+^·PF_6_^−^	*Z* = 2
*M**_r_* = 563.58	*F*(000) = 588
Triclinic, *P*1	*D*_x_ = 1.406 Mg m^−^^3^
Hall symbol: -P 1	Mo *K*α radiation, λ = 0.71073 Å
*a* = 9.0111 (14) Å	Cell parameters from 786 reflections
*b* = 9.1376 (15) Å	θ = 2.4–27.9°
*c* = 17.564 (3) Å	µ = 0.25 mm^−^^1^
α = 96.532 (3)°	*T* = 120 K
β = 100.225 (4)°	Block, colourless
γ = 108.053 (3)°	0.33 × 0.30 × 0.26 mm
*V* = 1331.0 (4) Å^3^	

### Data collection

**Table:**

Bruker SMART APEX diffractometer	6281 independent reflections
Radiation source: sealed tube	4547 reflections with *I* > 2σ(*I*)
graphite	*R*_int_ = 0.060
φ and ω scans	θ_max_ = 27.9°, θ_min_ = 2.4°
Absorption correction: multi-scan (*SADABS*; Sheldrick, 2004)	*h* = −11→10
*T*_min_ = 0.924, *T*_max_ = 0.939	*k* = −12→11
11922 measured reflections	*l* = −23→20

### Refinement

**Table:**

Refinement on *F*^2^	Primary atom site location: structure-invariant direct methods
Least-squares matrix: full	Secondary atom site location: difference Fourier map
*R*[*F*^2^ > 2σ(*F*^2^)] = 0.047	Hydrogen site location: difference Fourier map
*wR*(*F*^2^) = 0.104	H-atom parameters constrained
*S* = 0.96	*w* = 1/[σ^2^(*F*_o_^2^) + (0.0445*P*)^2^] where *P* = (*F*_o_^2^ + 2*F*_c_^2^)/3
6281 reflections	(Δ/σ)_max_ < 0.001
338 parameters	Δρ_max_ = 0.38 e Å^−^^3^
0 restraints	Δρ_min_ = −0.42 e Å^−^^3^

### Special details

**Table:**

Geometry. All e.s.d.'s (except the e.s.d. in the dihedral angle between two l.s. planes) are estimated using the full covariance matrix. The cell e.s.d.'s are taken into account individually in the estimation of e.s.d.'s in distances, angles and torsion angles; correlations between e.s.d.'s in cell parameters are only used when they are defined by crystal symmetry. An approximate (isotropic) treatment of cell e.s.d.'s is used for estimating e.s.d.'s involving l.s. planes.
Refinement. Refinement of *F*^2^ against ALL reflections. The weighted *R*-factor *wR* and goodness of fit *S* are based on *F*^2^, conventional *R*-factors *R* are based on *F*, with *F* set to zero for negative *F*^2^. The threshold expression of *F*^2^ > σ(*F*^2^) is used only for calculating *R*-factors(gt) *etc*. and is not relevant to the choice of reflections for refinement. *R*-factors based on *F*^2^ are statistically about twice as large as those based on *F*, and *R*-factors based on ALL data will be even larger.

### Fractional atomic coordinates and isotropic or equivalent isotropic displacement parameters (Å^2^)

**Table:**

	*x*	*y*	*z*	*U*_iso_*/*U*_eq_	
S1	0.77994 (6)	0.61888 (5)	0.29140 (3)	0.02002 (12)	
N1	0.6327 (2)	0.89606 (18)	0.43688 (9)	0.0242 (4)	
H1A	0.6289	0.8850	0.4857	0.029*	
N2	0.7383 (2)	1.16512 (18)	0.47245 (9)	0.0253 (4)	
N3	0.5695 (2)	1.05179 (18)	0.34970 (9)	0.0252 (4)	
C1	0.6470 (2)	1.0380 (2)	0.41911 (10)	0.0211 (4)	
C2	0.7029 (3)	1.3121 (2)	0.47981 (13)	0.0378 (6)	
H2A	0.7840	1.3922	0.4625	0.057*	
H2B	0.7042	1.3471	0.5348	0.057*	
H2C	0.5969	1.2948	0.4470	0.057*	
C3	0.8710 (3)	1.1626 (3)	0.53285 (12)	0.0338 (5)	
H3A	0.8341	1.1432	0.5812	0.051*	
H3B	0.9578	1.2636	0.5434	0.051*	
H3C	0.9102	1.0792	0.5145	0.051*	
C4	0.6343 (3)	1.1837 (3)	0.31065 (13)	0.0407 (6)	
H4A	0.5737	1.2556	0.3145	0.061*	
H4B	0.6252	1.1443	0.2551	0.061*	
H4C	0.7473	1.2390	0.3362	0.061*	
C5	0.4191 (3)	0.9342 (3)	0.30549 (12)	0.0350 (5)	
H5A	0.4396	0.8717	0.2619	0.052*	
H5B	0.3447	0.9862	0.2845	0.052*	
H5C	0.3720	0.8657	0.3403	0.052*	
C6	0.6227 (2)	0.7568 (2)	0.38190 (11)	0.0245 (4)	
H6A	0.5129	0.7109	0.3485	0.029*	
H6B	0.6430	0.6775	0.4124	0.029*	
C7	0.7419 (2)	0.7943 (2)	0.32952 (11)	0.0207 (4)	
H7A	0.8437	0.8738	0.3601	0.025*	
H7B	0.6989	0.8384	0.2852	0.025*	
C8	0.7037 (2)	0.5855 (2)	0.18294 (10)	0.0173 (4)	
C11	0.6980 (2)	0.4160 (2)	0.15696 (10)	0.0177 (4)	
C12	0.8390 (2)	0.3816 (2)	0.17641 (11)	0.0226 (4)	
H12A	0.9333	0.4611	0.2068	0.027*	
C13	0.8442 (2)	0.2340 (2)	0.15229 (12)	0.0269 (4)	
H13A	0.9412	0.2127	0.1664	0.032*	
C14	0.7079 (3)	0.1177 (2)	0.10767 (11)	0.0270 (5)	
H14A	0.7109	0.0164	0.0906	0.032*	
C15	0.5672 (3)	0.1497 (2)	0.08807 (11)	0.0266 (4)	
H15A	0.4732	0.0697	0.0578	0.032*	
C16	0.5622 (2)	0.2984 (2)	0.11234 (11)	0.0220 (4)	
H16A	0.4649	0.3192	0.0982	0.026*	
C21	0.5359 (2)	0.5994 (2)	0.16573 (10)	0.0172 (4)	
C22	0.4204 (2)	0.5180 (2)	0.20308 (10)	0.0206 (4)	
H22A	0.4475	0.4567	0.2399	0.025*	
C23	0.2669 (2)	0.5248 (2)	0.18760 (11)	0.0230 (4)	
H23A	0.1900	0.4685	0.2138	0.028*	
C24	0.2246 (2)	0.6135 (2)	0.13396 (11)	0.0237 (4)	
H24A	0.1192	0.6181	0.1233	0.028*	
C25	0.3372 (2)	0.6948 (2)	0.09638 (11)	0.0235 (4)	
H25A	0.3092	0.7558	0.0596	0.028*	
C26	0.4920 (2)	0.6881 (2)	0.11204 (11)	0.0208 (4)	
H26A	0.5686	0.7448	0.0858	0.025*	
C31	0.8208 (2)	0.6940 (2)	0.14281 (10)	0.0183 (4)	
C32	0.8071 (2)	0.6503 (2)	0.06202 (11)	0.0231 (4)	
H32A	0.7257	0.5565	0.0338	0.028*	
C33	0.9100 (2)	0.7415 (2)	0.02285 (11)	0.0268 (4)	
H33A	0.8989	0.7100	−0.0319	0.032*	
C34	1.0294 (2)	0.8785 (2)	0.06290 (12)	0.0259 (4)	
H34A	1.1011	0.9406	0.0361	0.031*	
C35	1.0432 (2)	0.9240 (2)	0.14238 (12)	0.0252 (4)	
H35A	1.1240	1.0185	0.1701	0.030*	
C36	0.9401 (2)	0.8329 (2)	0.18186 (11)	0.0216 (4)	
H36A	0.9512	0.8658	0.2365	0.026*	
P1	0.19146 (7)	0.27386 (6)	0.37421 (3)	0.02543 (13)	
F1	0.02793 (18)	0.13314 (18)	0.35865 (10)	0.0699 (5)	
F2	0.20703 (19)	0.22909 (16)	0.28687 (7)	0.0526 (4)	
F3	0.35707 (17)	0.41217 (16)	0.39103 (9)	0.0547 (4)	
F4	0.10033 (18)	0.38923 (16)	0.34893 (8)	0.0531 (4)	
F5	0.18070 (19)	0.31694 (17)	0.46304 (8)	0.0524 (4)	
F6	0.28644 (18)	0.15713 (16)	0.40077 (7)	0.0472 (4)	

### Atomic displacement parameters (Å^2^)

**Table:**

	*U*^11^	*U*^22^	*U*^33^	*U*^12^	*U*^13^	*U*^23^
S1	0.0229 (3)	0.0197 (2)	0.0182 (2)	0.0101 (2)	0.00173 (19)	0.00234 (17)
N1	0.0361 (10)	0.0218 (8)	0.0170 (8)	0.0095 (8)	0.0118 (7)	0.0047 (6)
N2	0.0275 (10)	0.0227 (8)	0.0245 (9)	0.0083 (7)	0.0052 (7)	0.0003 (7)
N3	0.0312 (10)	0.0246 (9)	0.0219 (8)	0.0126 (8)	0.0058 (7)	0.0043 (7)
C1	0.0222 (11)	0.0251 (10)	0.0198 (9)	0.0107 (8)	0.0100 (8)	0.0032 (8)
C2	0.0447 (15)	0.0216 (11)	0.0446 (14)	0.0110 (10)	0.0088 (11)	−0.0019 (9)
C3	0.0299 (12)	0.0375 (12)	0.0279 (11)	0.0083 (10)	0.0006 (10)	−0.0007 (9)
C4	0.0584 (17)	0.0377 (13)	0.0347 (12)	0.0214 (12)	0.0157 (12)	0.0186 (10)
C5	0.0346 (13)	0.0395 (13)	0.0291 (11)	0.0195 (11)	−0.0029 (10)	−0.0047 (9)
C6	0.0339 (12)	0.0191 (9)	0.0225 (10)	0.0103 (9)	0.0093 (9)	0.0031 (7)
C7	0.0222 (10)	0.0161 (9)	0.0232 (10)	0.0066 (8)	0.0045 (8)	0.0026 (7)
C8	0.0179 (10)	0.0160 (9)	0.0159 (9)	0.0045 (8)	0.0018 (7)	0.0019 (7)
C11	0.0207 (10)	0.0170 (9)	0.0168 (9)	0.0068 (8)	0.0064 (8)	0.0045 (7)
C12	0.0195 (10)	0.0198 (9)	0.0273 (10)	0.0057 (8)	0.0054 (8)	0.0022 (8)
C13	0.0281 (12)	0.0250 (10)	0.0328 (11)	0.0137 (9)	0.0100 (9)	0.0067 (8)
C14	0.0374 (13)	0.0169 (9)	0.0292 (11)	0.0108 (9)	0.0122 (10)	0.0018 (8)
C15	0.0295 (12)	0.0208 (10)	0.0234 (10)	0.0012 (9)	0.0066 (9)	−0.0008 (8)
C16	0.0215 (11)	0.0224 (10)	0.0204 (9)	0.0069 (8)	0.0026 (8)	0.0020 (7)
C21	0.0168 (10)	0.0149 (8)	0.0173 (9)	0.0054 (7)	−0.0001 (7)	−0.0014 (7)
C22	0.0227 (11)	0.0200 (9)	0.0211 (10)	0.0091 (8)	0.0044 (8)	0.0065 (7)
C23	0.0192 (10)	0.0227 (10)	0.0270 (10)	0.0055 (8)	0.0068 (8)	0.0061 (8)
C24	0.0173 (10)	0.0258 (10)	0.0287 (11)	0.0100 (8)	0.0033 (8)	0.0033 (8)
C25	0.0248 (11)	0.0229 (10)	0.0250 (10)	0.0115 (9)	0.0029 (9)	0.0077 (8)
C26	0.0210 (10)	0.0191 (9)	0.0230 (10)	0.0070 (8)	0.0052 (8)	0.0057 (7)
C31	0.0173 (10)	0.0179 (9)	0.0228 (9)	0.0094 (8)	0.0046 (8)	0.0052 (7)
C32	0.0210 (10)	0.0216 (9)	0.0246 (10)	0.0057 (8)	0.0023 (8)	0.0044 (8)
C33	0.0290 (12)	0.0313 (11)	0.0226 (10)	0.0114 (10)	0.0068 (9)	0.0098 (8)
C34	0.0246 (11)	0.0262 (10)	0.0336 (11)	0.0122 (9)	0.0110 (9)	0.0155 (9)
C35	0.0190 (10)	0.0175 (9)	0.0375 (12)	0.0047 (8)	0.0041 (9)	0.0061 (8)
C36	0.0197 (10)	0.0200 (9)	0.0253 (10)	0.0078 (8)	0.0044 (8)	0.0033 (8)
P1	0.0307 (3)	0.0207 (3)	0.0231 (3)	0.0066 (2)	0.0047 (2)	0.0047 (2)
F1	0.0451 (10)	0.0428 (9)	0.0994 (13)	−0.0102 (7)	0.0113 (9)	0.0027 (9)
F2	0.0916 (12)	0.0495 (9)	0.0248 (7)	0.0340 (9)	0.0148 (7)	0.0079 (6)
F3	0.0403 (9)	0.0380 (8)	0.0720 (10)	−0.0026 (7)	0.0121 (8)	0.0015 (7)
F4	0.0661 (11)	0.0509 (9)	0.0512 (9)	0.0377 (8)	0.0019 (8)	0.0112 (7)
F5	0.0803 (11)	0.0580 (9)	0.0337 (7)	0.0362 (9)	0.0256 (7)	0.0098 (6)
F6	0.0765 (11)	0.0483 (8)	0.0350 (7)	0.0411 (8)	0.0162 (7)	0.0173 (6)

### Geometric parameters (Å, °)

**Table:**

S1—C7	1.8202 (17)	C13—C14	1.383 (3)
S1—C8	1.8620 (18)	C13—H13A	0.9500
N1—C1	1.341 (2)	C14—C15	1.381 (3)
N1—C6	1.476 (2)	C14—H14A	0.9500
N1—H1A	0.8800	C15—C16	1.394 (2)
N2—C1	1.338 (2)	C15—H15A	0.9500
N2—C3	1.459 (3)	C16—H16A	0.9500
N2—C2	1.470 (2)	C21—C22	1.393 (3)
N3—C1	1.333 (2)	C21—C26	1.394 (2)
N3—C5	1.459 (3)	C22—C23	1.384 (3)
N3—C4	1.469 (3)	C22—H22A	0.9500
C2—H2A	0.9800	C23—C24	1.388 (3)
C2—H2B	0.9800	C23—H23A	0.9500
C2—H2C	0.9800	C24—C25	1.378 (3)
C3—H3A	0.9800	C24—H24A	0.9500
C3—H3B	0.9800	C25—C26	1.395 (3)
C3—H3C	0.9800	C25—H25A	0.9500
C4—H4A	0.9800	C26—H26A	0.9500
C4—H4B	0.9800	C31—C36	1.390 (2)
C4—H4C	0.9800	C31—C32	1.402 (2)
C5—H5A	0.9800	C32—C33	1.381 (3)
C5—H5B	0.9800	C32—H32A	0.9500
C5—H5C	0.9800	C33—C34	1.384 (3)
C6—C7	1.521 (3)	C33—H33A	0.9500
C6—H6A	0.9900	C34—C35	1.383 (3)
C6—H6B	0.9900	C34—H34A	0.9500
C7—H7A	0.9900	C35—C36	1.385 (3)
C7—H7B	0.9900	C35—H35A	0.9500
C8—C21	1.537 (2)	C36—H36A	0.9500
C8—C31	1.538 (2)	P1—F4	1.5801 (13)
C8—C11	1.546 (2)	P1—F3	1.5814 (14)
C11—C16	1.386 (3)	P1—F1	1.5819 (15)
C11—C12	1.396 (3)	P1—F2	1.5842 (13)
C12—C13	1.385 (2)	P1—F5	1.5924 (13)
C12—H12A	0.9500	P1—F6	1.6202 (13)
			
C7—S1—C8	105.13 (8)	C14—C13—H13A	120.1
C1—N1—C6	125.84 (15)	C12—C13—H13A	120.1
C1—N1—H1A	117.1	C15—C14—C13	119.59 (17)
C6—N1—H1A	117.1	C15—C14—H14A	120.2
C1—N2—C3	122.00 (16)	C13—C14—H14A	120.2
C1—N2—C2	122.76 (17)	C14—C15—C16	120.53 (18)
C3—N2—C2	114.95 (16)	C14—C15—H15A	119.7
C1—N3—C5	122.70 (17)	C16—C15—H15A	119.7
C1—N3—C4	121.96 (18)	C11—C16—C15	120.46 (18)
C5—N3—C4	115.28 (17)	C11—C16—H16A	119.8
N3—C1—N2	120.65 (17)	C15—C16—H16A	119.8
N3—C1—N1	120.34 (17)	C22—C21—C26	117.89 (17)
N2—C1—N1	119.01 (17)	C22—C21—C8	119.67 (15)
N2—C2—H2A	109.5	C26—C21—C8	122.42 (17)
N2—C2—H2B	109.5	C23—C22—C21	121.25 (17)
H2A—C2—H2B	109.5	C23—C22—H22A	119.4
N2—C2—H2C	109.5	C21—C22—H22A	119.4
H2A—C2—H2C	109.5	C22—C23—C24	120.33 (18)
H2B—C2—H2C	109.5	C22—C23—H23A	119.8
N2—C3—H3A	109.5	C24—C23—H23A	119.8
N2—C3—H3B	109.5	C25—C24—C23	119.29 (18)
H3A—C3—H3B	109.5	C25—C24—H24A	120.4
N2—C3—H3C	109.5	C23—C24—H24A	120.4
H3A—C3—H3C	109.5	C24—C25—C26	120.40 (17)
H3B—C3—H3C	109.5	C24—C25—H25A	119.8
N3—C4—H4A	109.5	C26—C25—H25A	119.8
N3—C4—H4B	109.5	C21—C26—C25	120.84 (18)
H4A—C4—H4B	109.5	C21—C26—H26A	119.6
N3—C4—H4C	109.5	C25—C26—H26A	119.6
H4A—C4—H4C	109.5	C36—C31—C32	117.81 (17)
H4B—C4—H4C	109.5	C36—C31—C8	123.97 (16)
N3—C5—H5A	109.5	C32—C31—C8	118.22 (16)
N3—C5—H5B	109.5	C33—C32—C31	121.00 (18)
H5A—C5—H5B	109.5	C33—C32—H32A	119.5
N3—C5—H5C	109.5	C31—C32—H32A	119.5
H5A—C5—H5C	109.5	C32—C33—C34	120.39 (18)
H5B—C5—H5C	109.5	C32—C33—H33A	119.8
N1—C6—C7	112.59 (15)	C34—C33—H33A	119.8
N1—C6—H6A	109.1	C35—C34—C33	119.25 (18)
C7—C6—H6A	109.1	C35—C34—H34A	120.4
N1—C6—H6B	109.1	C33—C34—H34A	120.4
C7—C6—H6B	109.1	C34—C35—C36	120.53 (18)
H6A—C6—H6B	107.8	C34—C35—H35A	119.7
C6—C7—S1	110.58 (12)	C36—C35—H35A	119.7
C6—C7—H7A	109.5	C35—C36—C31	121.02 (18)
S1—C7—H7A	109.5	C35—C36—H36A	119.5
C6—C7—H7B	109.5	C31—C36—H36A	119.5
S1—C7—H7B	109.5	F4—P1—F3	90.03 (8)
H7A—C7—H7B	108.1	F4—P1—F1	91.33 (9)
C21—C8—C31	112.93 (14)	F3—P1—F1	178.58 (9)
C21—C8—C11	111.65 (14)	F4—P1—F2	91.47 (8)
C31—C8—C11	108.14 (14)	F3—P1—F2	89.85 (8)
C21—C8—S1	108.93 (12)	F1—P1—F2	90.54 (9)
C31—C8—S1	112.31 (12)	F4—P1—F5	90.31 (8)
C11—C8—S1	102.41 (11)	F3—P1—F5	89.46 (8)
C16—C11—C12	118.30 (16)	F1—P1—F5	90.11 (9)
C16—C11—C8	123.45 (16)	F2—P1—F5	178.08 (8)
C12—C11—C8	118.19 (16)	F4—P1—F6	179.38 (9)
C13—C12—C11	121.31 (18)	F3—P1—F6	89.43 (8)
C13—C12—H12A	119.3	F1—P1—F6	89.21 (9)
C11—C12—H12A	119.3	F2—P1—F6	88.82 (7)
C14—C13—C12	119.82 (18)	F5—P1—F6	89.39 (7)
			
C5—N3—C1—N2	−150.95 (18)	C14—C15—C16—C11	−0.4 (3)
C4—N3—C1—N2	32.1 (3)	C31—C8—C21—C22	−174.76 (15)
C5—N3—C1—N1	28.1 (3)	C11—C8—C21—C22	63.1 (2)
C4—N3—C1—N1	−148.85 (18)	S1—C8—C21—C22	−49.25 (19)
C3—N2—C1—N3	−154.99 (18)	C31—C8—C21—C26	6.9 (2)
C2—N2—C1—N3	31.5 (3)	C11—C8—C21—C26	−115.21 (18)
C3—N2—C1—N1	25.9 (3)	S1—C8—C21—C26	132.42 (15)
C2—N2—C1—N1	−147.63 (19)	C26—C21—C22—C23	0.0 (3)
C6—N1—C1—N3	37.8 (3)	C8—C21—C22—C23	−178.44 (16)
C6—N1—C1—N2	−143.12 (19)	C21—C22—C23—C24	0.1 (3)
C1—N1—C6—C7	43.2 (3)	C22—C23—C24—C25	−0.1 (3)
N1—C6—C7—S1	158.36 (13)	C23—C24—C25—C26	0.0 (3)
C8—S1—C7—C6	115.92 (14)	C22—C21—C26—C25	0.0 (3)
C7—S1—C8—C21	−47.42 (14)	C8—C21—C26—C25	178.32 (16)
C7—S1—C8—C31	78.45 (13)	C24—C25—C26—C21	0.1 (3)
C7—S1—C8—C11	−165.77 (12)	C21—C8—C31—C36	103.47 (19)
C21—C8—C11—C16	9.8 (2)	C11—C8—C31—C36	−132.46 (17)
C31—C8—C11—C16	−115.03 (19)	S1—C8—C31—C36	−20.2 (2)
S1—C8—C11—C16	126.21 (16)	C21—C8—C31—C32	−76.83 (19)
C21—C8—C11—C12	−173.28 (15)	C11—C8—C31—C32	47.2 (2)
C31—C8—C11—C12	61.9 (2)	S1—C8—C31—C32	159.51 (14)
S1—C8—C11—C12	−56.87 (18)	C36—C31—C32—C33	0.7 (3)
C16—C11—C12—C13	−0.3 (3)	C8—C31—C32—C33	−179.02 (16)
C8—C11—C12—C13	−177.34 (17)	C31—C32—C33—C34	0.1 (3)
C11—C12—C13—C14	0.4 (3)	C32—C33—C34—C35	−0.8 (3)
C12—C13—C14—C15	−0.5 (3)	C33—C34—C35—C36	0.8 (3)
C13—C14—C15—C16	0.5 (3)	C34—C35—C36—C31	0.0 (3)
C12—C11—C16—C15	0.3 (3)	C32—C31—C36—C35	−0.7 (3)
C8—C11—C16—C15	177.21 (17)	C8—C31—C36—C35	178.97 (16)

### Hydrogen-bond geometry (Å, °)

**Table:**

*D*—H···*A*	*D*—H	H···*A*	*D*···*A*	*D*—H···*A*
N1—H1A···F6^i^	0.88	2.13	2.949 (2)	155.

Symmetry codes: (i) −*x*+1, −*y*+1, −*z*+1.
